# An evaluation of enteral nutrition practices and nutritional provision in children during the entire length of stay in critical care

**DOI:** 10.1186/1471-2431-14-186

**Published:** 2014-07-21

**Authors:** Jackie Mara, Emma Gentles, Hani A Alfheeaid, Krystalia Diamantidi, Neil Spenceley, Mark Davidson, David Young, Konstantinos Gerasimidis

**Affiliations:** 1 Paediatric Intensive Care Unit, Royal Hospital for Sick Children, NHS Greater Glasgow and Clyde, Glasgow G3 8SJ, UK; 2 Department of Dietetics and Nutrition, Royal Hospital for Sick Children, NHS Greater Glasgow and Clyde, Glasgow G3 8SJ, UK; 3 Human Nutrition, School of Medicine, College of Medicine, Veterinary and Life Sciences, University of Glasgow, Glasgow G3 8SJ, UK; 4 Department of Mathematics and Statistics, University of Strathclyde, Glasgow G1 1XH, UK

**Keywords:** Paediatric intensive care unit, Critical care, Enteral nutrition, Nutrition support

## Abstract

**Background:**

Provision of optimal nutrition in children in critical care is often challenging. This study evaluated exclusive enteral nutrition (EN) provision practices and explored predictors of energy intake and delay of EN advancement in critically ill children.

**Methods:**

Data on intake and EN practices were collected on a daily basis and compared against predefined targets and dietary reference values in a paediatric intensive care unit. Factors associated with intake and advancement of EN were explored.

**Results:**

Data were collected from 130 patients and 887 nutritional support days (NSDs). Delay to initiate EN was longer in patients from both the General Surgical and congenital heart defect (CHD) Surgical groups [Median (IQR); CHD Surgical group: 20.3 (16.4) vs General Surgical group: 11.4 (53.5) vs Medical group: 6.5 (10.9) hours; p ≤ 0.001]. Daily fasting time per patient was significantly longer in patients from the General Surgical and CHD Surgical groups than those from the Medical group [% of 24 h, Median (IQR); CHD Surgical group: 24.0 (29.2) vs General Surgical group: 41.7 (66.7) vs Medical group: 9.4 (21.9); p ≤ 0.001]. A lower proportion of fluids was delivered as EN per patient (45% vs 73%) or per NSD (56% vs 73%) in those from the CHD Surgical group compared with those with medical conditions. Protein and energy requirements were achieved in 38% and 33% of the NSDs. In a substantial proportion of NSDs, minimum micronutrient recommendations were not met particularly in those patients from the CHD Surgical group. A higher delivery of fluid requirements (p < 0.05) and a greater proportion of these delivered as EN (p < 0.001) were associated with median energy intake during stay and delay of EN advancement. Fasting (31%), fluid restriction (39%) for clinical reasons, procedures requiring feed cessation and establishing EN (22%) were the most common reasons why target energy requirements were not met.

**Conclusions:**

Provision of optimal EN support remains challenging and varies during hospitalisation and among patients. Delivery of EN should be prioritized over other “non-nutritional” fluids whenever this is possible.

## Background

A substantial number of children in critical care are malnourished on admission and a proportion of them will deteriorate due to the metabolic response to injury, surgery or inflammation [1,2]. Although nutritional support is unlikely to reverse the course of illness, optimal nutritional support can minimize nutrient deficits and delay establishment of malnutrition, thereby potentially improving the clinical outcome of the patient [3].

Thus, provision of optimal nutrition is central for the health and disease prognosis of the critically ill child and should be an integral part of any service aiming to provide optimal care. However this is not always easy to achieve as the clinical team frequently encounters a number of barriers to the estimation and delivery of nutritional support in the paediatric intensive care unit (PICU) [4]. These include the estimation rather than measurement of nutritional needs of the individual child, under-prescription and inadequate delivery of nutrients owing to strict fluid volume monitoring, interruptions or cessation of nutritional support due to gastrointestinal intolerance or mechanical problems, but also lack of nutritional awareness and routine assessment of patients [4]. Thus, several paediatric intensive care units have reported their experience of improving the delivery of nutritional support and its impact on clinical outcomes by implementation of nutritional management protocols and guidelines [5-7].

Despite the ongoing debate on the impact of early nutritional support on clinical outcomes, such as reduction of mortality, invasive ventilation and length of hospital stay [8-10], in current practice every effort is given to initiate early feeding and to improve the delivery of nutritional requirements using enteral nutrition (EN), limiting whenever possible use of the parenteral route. The effectiveness of the nutritional adequacy of exclusive EN remains unclear and may vary according to the presence or not, of multidisciplinary management and dietetic support.

Although there is substantial evidence to describe nutritional practices and provision in children admitted to PICU [11,12] there are limited data to explore such aspects prospectively over the entire duration of hospital stay and to study predictors associated with initiation, advancement and establishment of nutritional support [11,13]. Identifying modifiable barriers of nutritional provision and windows for improvement will allow the clinical team to intervene timely and adopt the optimal management plan which will have the maximum possible benefit to the nutritional support and potentially clinical outcome of the sick child in critical care.

We studied EN support practices and energy/nutrient provision during the entire length of stay in a PICU and explored factors associated with energy intake and successful advancement of EN.

## Methods

This study took place in a 22 bed mixed speciality PICU at the Royal Hospital for Sick Children, Glasgow, United Kingdom. Two cohorts of participants were included; one between 1^st^ January to 30^th^ March 2009 and a second one in the same period a year later. All patients with a PICU length of stay of more than 48 hours and who were fed exclusively with EN were included. Children who received partial or total parental nutrition support or oral diet during admission were excluded in order to minimise heterogeneity in our nutritional support modalities as well as to explore whether using exclusive EN would allow delivery of adequate energy/nutrient requirements. At the time of this study there were two PICU specialist dietitians, a prescription pharmacist, and a senior critical care nutrition specialist nurse allocated to the unit. Patients were referred to dietitians either on clinician’s request or according to local clinical management pathways.

Information on patients’ disease characteristics were recorded from electronic records. Clinical conditions were classified into three diagnostic groups: Medical (those admitted for non-surgical reasons), Congenital Heart Disease (CHD) Surgical (those admitted after corrective heart surgery) and General Surgical (those admitted after undergoing any surgery other than corrective heart surgery). EN support practices and nutritional intake were collected on a daily basis from the unit Computerised Information System (CIS, Metavision, iMDsoft®, Woking, United Kingdom). These included: route of EN administration, time elapsed from PICU admission to initiation of EN, daily fasting time, enteral feed composition and total daily intravenous fluid and EN volume administered. Data were recorded from the time of PICU admission and were collected prospectively for each complete 24 hour period of admission (nutritional support day-NSD) and until discharge. Incomplete data from the last day of PICU stay were excluded. During the first period we also collected data on barriers of achieving minimal energy requirements. This was not possible due to logistical reasons in the second period.

In the absence of continuous monitoring of energy expenditure with indirect calorimetry, the assumption was made that patients’ energy requirements were equal to those of the basal metabolic rate (BMR) for healthy children using the Schofield equations [14], with no correction for stress factors [15]. This is common practice in UK and other hospitals around the world. Fluid requirements were calculated based on body weight [16]. Patients’ daily intake of protein was expressed as a percentage of Reference Nutrient Intake (%RNI) while the intake of micronutrients was classified as above or below the Lower Reference Nutrient Intake (LRNI) [17]. Data on EN support practices and nutritional provision were presented in two ways: a) median intake per patient during the entire PICU stay and b) median intake per NSD.

Weight measurements were converted to z-score based on the UK 1990 reference data corrected for gestational age [18] and underweight was defined as a weight z-score equal or below −2 SD.

### Statistical analysis

Continuous variables were expressed as medians with inter-quartile ranges and analysed with non-parametric statistics (Kruskal-Wallis, Mann–Whitney tests) for differences between groups. Categorical data were presented with counts and percentages and differences between groups were explored with Chi-squared test or Fisher’s exact test.

Factors predicting median energy intake (expressed as% of BMR) during the duration of stay were explored with univariate and multivariate (predictors with p < 0.1 were entered in the model) stepwise linear regression analysis. Predictors set *a priori* and included: age, prematurity, weight z-score, diagnostic group, duration of stay in PICU, time elapsed from admission to initiate EN, median daily fasting time (%) during hospitalization, percent of fluid requirements delivered, fluid requirements delivered as EN (%) and PIM2 (Paediatric Index of Mortality) score as prognostic index of mortality which is computed using clinical information collected at the time of admission to PICU [19].

Similarly, delay to advance EN (i.e. number of days elapsed between admission to provision of energy requirements equal to BMR) was explored with univariate survival analysis on each predictor using Cox regression analysis for quantitative variables and Log-rank test for categorical variables. Variables which were significant at the 5% level univariately, were used in a stepwise multivariate Cox regression model to determine which were independently predictive of the time to achieve full nutritional requirements.

Statistical analysis was performed with MINITAB version 16 (Minitab Ltd) and SPSS version 21 at a 5% significance level.

### Ethics approval

The study was registered with the local Clinical Effectiveness Department as a study auditing current clinical practice.

## Results

### Patients’ characteristics

In total 130 patients were eligible and included. Twenty eight (18%) others who received parenteral nutrition were excluded. Children in the CHD Surgical and Medical groups were significantly younger than those from the General Surgical group (Table 1). Median weight z-score was significantly lower in patients with CHD compared with the General Surgical and Medical groups, and a third of this group were underweight as compared with approximately 15% and 18% of patients admitted with medical conditions and for general surgical reasons respectively (Table 1). Those in the CHD Surgical group had a significantly higher median PIM2 score than those from the General Surgical group [PIM2: Median (IQR); CHD Surgical group: 2.3 (3.1) vs General Surgical group: 0.5 (4.2); p = 0.027] (Table 1). There was a trend towards the CHD Surgical group having a higher PIM2 score than the Medical group (p = 0.067) and similarly for the latter compared with the General Surgical group (p = 0.062) (Table 1). Three children died during the study period.

**Table 1:** Demographics, anthropometry and disease characteristics of children admitted in a paediatric intensive care unit

**Diagnostic group**	**CHD surgical**		**Medical**		**General surgical**	
	**n = 48**		**n = 71**		**n = 11**	
	**Median/N**	**IQR/%**	**Median/N**	**IQR/%**	**Median/N**	**IQR/%**
NSDs	367		453		67	
Corrected age (y)	0.3^*^	0.9	0.3^**^	1.8	4.9	11.3
Age <1 y	37	77%	44	62%	3	27%
Gender						
Male	32	67%	37	52%	6	54%
Female	16	33%	34	48%	5	45%
Premature	9	19%	11	15%	3	27%
Weight (kg)	4.9	4.3	6.2	7.8	22.8	23.3
Weight z-score (SD)	−1.5^***^	1.4	−0.3	1.9	−0.8	2.0
Weight z-score ≤ −2	15	31%	11	15%	2	18%
PIM2	2.3^*^	3.1	1.2	3.9	0.5	4.2
Ventilation time (h)	93.5	115.5	106.0	100.0	72.0	197.0
PICU LOS (d)	5.8	5.7	5.7	4.6	5.7	5.3
Total LOS (d)	18.0^***^	25.8	11.0	12.0	12.0	25.0
Deceased	1	2.1%	2	2.8%	0	0%

^*^CHD Surgical significantly different from General Surgical; ^**^Medical different from General Surgical; ^***^CHD Surgical significantly different from Medical; NSDs: Nutritional Support Days; PIM2: Paediatric Index of Mortality score significantly; PICU LOS: Length of stay in paediatric intensive care unit (PICU), Total LOS: Total length of stay in hospital (i.e. ward and intensive care unit) per patient; IQR: Interquartile range.

### Enteral nutrition practices

The majority of the patients were fed via a nasogastric tube. Five patients received EN via a nasojejunal tube due to increased (two consecutive 4-hourly measured gastric residual volumes > 5 ml/kg) gastric residual volumes (Table 2). High energy and elemental composition feeds were used in 14% and 9.5% of the NSDs respectively. Ninety nine (76%) patients received EN support within 24 hours of admission to PICU. Three patients did not receive any form of nutritional support for the entire duration of stay in the PICU (range of length of hospital stay 3 to 6 days).

A significantly lower proportion of patients from the CHD and General Surgical groups started EN support within 24 hours of admission than patients admitted with medical conditions [CHD Surgical group: 60% vs General Surgical group: 55% vs Medical group: 90%; p ≤ 0.001] (Table 2). Delay to initiate EN was significantly longer in patients from both the General Surgical and CHD Surgical groups compared to the Medical group [Median (IQR); CHD Surgical group: 20.3 (16.4) vs General Surgical group: 11.4 (53.5) vs Medical group: 6.5 (10.9) hours; p ≤ 0.001] (Table 2). Similarly the median daily fasting time per patient (not including the time to initiate EN) was significantly longer in patients from the General Surgical and CHD Surgical groups than those from the Medical group [% of 24 h, Median (IQR); CHD Surgical group: 24.0 (29.2) vs General Surgical group: 41.7 (66.7) vs Medical group: 9.4 (21.9); p ≤ 0.001] (Table 2).

**Table 2:** Enteral nutrition practices and nutritional intake in children admitted in a paediatric intensive care unit by diagnostic group

**Diagnostic group**	**CHD surgical**		**Medical group**		**General surgical**	
**N (patients)**	**n = 48**		**n = 71**		**n = 11**	
	**Median/N**	**IQR/%**	**Median/N**	**IQR/%**	**Median/N**	**IQR/%**
Time to start EN (h)	20.3^*^	16.4	6.5^***^	10.9	11.4	53.5
EN start <24 h	29^*^	60%	64^***^	90%	6	55%
Nasojejunal feeding (d)	2	4.2%	3	4.2%	0	0%
% Daily fasting	24.0^*^	29.2	9.4^***^	21.9	41.7	66.7
% Fluid requirements	65.3^**^	15.2	64.6^***^	24.1	86.8	25.4
% Total fluid as EN	44.8^*^	34.8	73.3	25.4	31.9	80.7
Energy (% BMR)	53.2^*^	47.8	87.6	42.2	42.8	119.7
Protein (% RNI)	34.7^*^	57.7	56.2	60.8	54.6	129.1
**N (NSDs)**	**N = 367**		**N = 453**		**N = 67**	
	**Median/N**	**IQR/%**	**Median/N**	**IQR/%**	**Median/N**	**IQR/%**
Nasojejunal feeding (d)	16	4.3%	21	4.6%	0	0%
% Daily fasting	14.6^*,**^	43.8	4.2^***^	33.3	29.2	95.8
% Fluid requirements	66.7^**^	23.1	66.7^***^	25.3	94.1	28.8
% Total fluid as EN	56.3^*^	56.2	72.7^***^	39.8	38.6	85.6
Energy (% BMR)	73.3^*^	83.7	88.2^***^	60.2	55.9	131.6
Protein (%RNI)	48.4^*^	78.7	60.0^***^	72.8	48.8	124.4

^*^CHD Surgical significantly different from Medical; ^**^CHD Surgical significantly different from general Surgical; ^***^Medical different from general Surgical; % Daily Fasting: Daily fasting hours expressed as percentage of 24 hours (excluding time to initiate feeding on admission); % Fluid Requirements: Percentage of daily fluid requirements delivered; % Total Fluid as EN: Percentage of total fluid volume delivered as enteral nutrition; BMR: Basal Metabolic Rate; RNI: Recommended Nutrient Intake; IQR: Interquartile range.

The volume of daily fluid delivered (% of requirements) per patient or per NSD were significantly lower in CHD Surgical patients and those admitted with medical conditions than those from the General Surgical group (Table 2). However the percentage of total daily fluid delivered as EN per NSD was lower in the General Surgical group when compared to the General Medical group (Table 2). A lower proportion of total fluid intake was delivered as EN per patient or per NSD in those from the CHD Surgical group compared with those admitted with medical conditions (Table 2).

### Energy and nutrient intake in PICU

**Figure 1:**
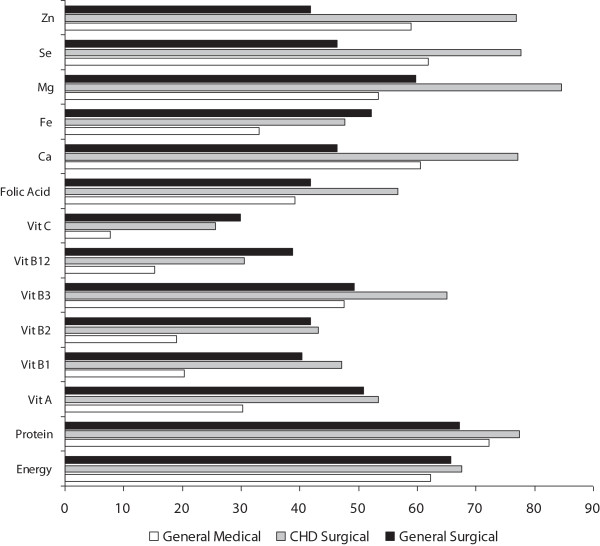
Proportion (%) of nutritional support days where daily requirements for energy (BMR), protein (RNI) and micronutrient (LRNI) were not achieved by diagnostic group. BMR: Basal metabolic rate; RNI: Recommended nutrient intake; LRNI: Lowest recommended nutrient intake; CHD: congenital heart defects.

**Figure 2:**
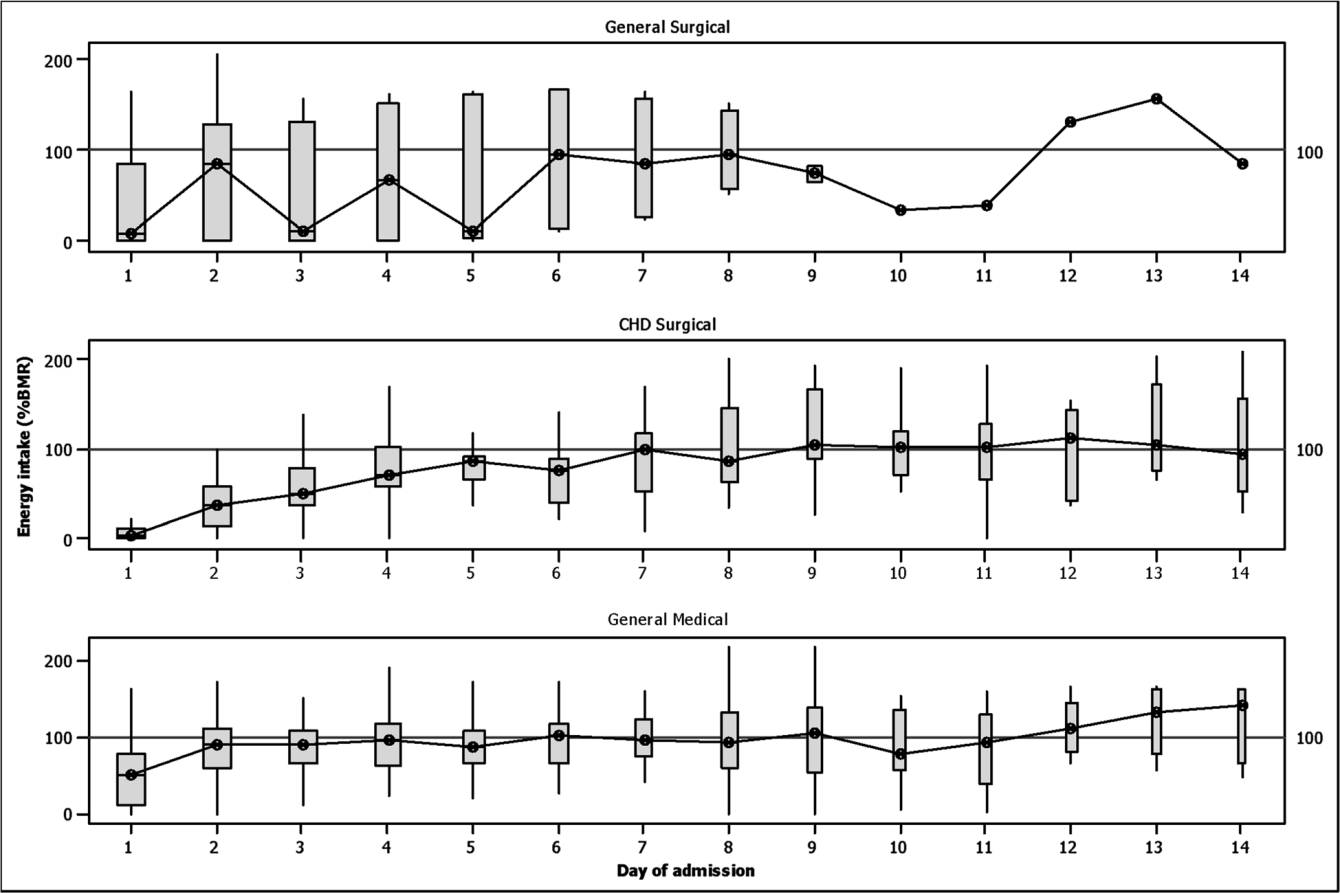
**Box plots of energy intake during stay in a paediatric intensive care unit (truncated to 14 days) by diagnostic group.** The width of the box is proportional to the number of measurements at each day of hospital stay.

The median daily intakes of energy (%BMR) and protein (%RNI) were significantly lower in the CHD Surgical patients compared with the General Medical group (Table 2). Energy requirements equal to BMR and protein requirements equal to RNI were not achieved in more than 62% and 67% of the NSDs respectively with no significant differences between the diagnostic groups (Figure 1). Ninety two percent of the patients received less than 100% of their BMR requirements at the first day of admission compared with 75% and 71% at the end of the second and third day respectively. Among the diagnostic groups, energy intake was worse for the CHD Surgical group (Figure 2). Minimum nutrient requirements (LRNI) were not achieved for a substantial number of NSD and for the large majority of micronutrients studied (Figure 1). In a higher proportion of NSD minimum micronutrient recommendations were achieved in the Medical and General Surgical groups compared to the CHD Surgical (Figure 1) but this varied and a particular pattern was not uniform across the individual micronutrients studied (Figure 1).

### Predictors of energy intake and delay of EN advancement

Median energy intakes per day of admission and diagnostic group are displayed in Figure 2. Independent predictors of median daily energy intake (%BMR) and delay of EN advancement were explored for the entire cohort of participants. A higher delivery of fluid requirements and a greater proportion of these delivered as EN, were independently associated with median energy intake during PICU stay and delay of EN advancement (Table 3). Similarly, child’s age was negatively associated with median energy intake in multivariate analysis (Table 3). These three factors explained 85% of the variation in median daily energy intake.

**Table 3:** Predictors of median energy intake during stay and delay of initial exclusive enteral nutrition advancement in a paediatric intensive care unit

	**Delay of EN advancement**		**Median energy intake**	
**Univariate**	**Hazard ratio**	**p-value**	**Coefficient**	**p-value**
	**[95% CI]**			
Age (y)	0.97 [0.90: 1.04]	0.410	-2.07	0.088
Prematurity	0.85 [0.63: 1.16]	0.311	-0.10	0.984
Weight z-score (SD)	0.96 [0.84: 1.11]	0.611	0.76	0.744
Diagnosis				
*Medical vs CHD*	1.74 [1.08: 2.79]	0.022	18.3	0.001
*Surgical vs CHD*	0.97 [0.39: 2.38]	0.945	-7.1	0.401
PICU LOS (d)	0.99 [0.97: 1.02]	0.739	1.16	0.021
Delay to initiate EN (h)			-0.98	<0.001
% Daily fasting	0.98 [0.96: 0.99]	0.001	-1.17	<0.001
% Fluid requirements	1.01 [1.00: 1.02]	0.046	0.46	0.025
% Total fluid as EN	1.03 [1.02: 1.04]	<0.001	1.40	<0.001
PIM2	0.97 [0.93: 1.01]	0.163	-0.76	0.023
**Multivariate**				
% Total fluid as EN	1.03 [1.02: 1.04]	<0.001	1.36	<0.001
Age (d)			-2.26	<0.001
% Fluid requirements	1.02 [1.00: 1.03]	0.004	0.73	<0.001

Delay of EN advancement: Days to achieve energy requirements equal of basal metabolic rate (BMR); Median energy intake: Median energy intake (%BMR) during the entire length of stay in Paediatric Intensive Care Unit (PICU). Delay to initiate EN (h): Time elapsed from admission to PICU to initiation of enteral nutrition; % Daily Fasting: Daily fasting hours expressed as percentage of 24 hours (excluding time to initiate feeding on admission); % Fluid Requirements: Percentage of daily fluid requirements delivered; % Total Fluid as EN: Percentage of total fluid volume delivered as enteral nutrition; PIM2: Paediatric Index of Mortality score; PICU LOS: Length of stay in PICU.

### Reasons of failing to achieve energy requirements

In the first period of the study, reasons for failing to achieve energy requirements were collected on daily basis. From the 477 NSDs, energy requirements equal to BMR were not delivered in 338 (71%). This was due to fasting (n = 104, 31%) and fluid restriction (n = 132, 39%) for clinical reasons, procedures being undertaken in the ward and establishing enteral feeding (n = 74, 22%). For the remaining 27 (8%) NSDs this was due to other reasons (e.g. raised gastric residual volumes, abdominal distension).

## Discussion

The results of this study highlight that under current multidisciplinary management delivery of minimal estimated requirements using exclusive EN was not optimal with the majority of patients achieving energy requirements lower than BMR and nutrient intakes lower than the minimal dietary references. This was particularly evident in the CHD Surgical group, where a substantial proportion of patients were already underweight on admission. The results of this study are similar to previous literature in children in critical care although a direct comparison is difficult due to differences in methodological aspects among the studies. Taylor *et al.* showed a median delivery of 60% of predefined targets during hospitalisation [20], de Oliveira Inglesias *et al.* reported that prescription and delivery of energy were not adequate in > 50% of enteral nutrition support days [12] and de Neef *et al.* found large inter-individual variations in the energy and nutrient intake during the first 10 days of admission [11].

In this study we explored predictors associated with nutritional delivery and speed of advancement of EN. Although disease diagnosis was a strong predictor in the univariate analysis this association was confounded with other potential determinants. The amount of fluid administered and a larger fraction of this delivered as EN were the strongest independent predictors.

Previous studies have also highlighted fluid restriction, fasting prior to clinical procedures and intolerance to EN support as primary reasons of failing to achieve optimal nutritional support in critically ill paediatric patients [11-13]. Indeed our findings are in agreement with those by Rogers *et al.* in Australia, where restriction of fluid intake was the main barrier to the delivery of adequate nutrition, particularly in infants undergoing cardiac surgery [21].

The current findings are in accordance with this evidence and suggest that fluid requirements should be optimised whenever possible and this should be done via nutritionally rich fluids in the form of EN or parenteral nutrition. This may be particularly important in fluid restricted patients where nutritional requirements are difficult to achieve. In the current study, the children with CHD were largely malnourished on hospital admission and were more likely to be sicker. In this nutritionally vulnerable group of children, with increased energy and nutrient demands [22], use of high energy feeds may be another option to consider improving nutritional intake until delivery of fluid and nutritional support becomes more liberal.

It has been previously shown that early initiation of feeding may improve nutritional delivery and implementation of local management protocols may facilitate this process [6]. Although it could be suggested that patients who cannot tolerate EN should be supplemented with PN this may not be appropriate in all patients particularly those who can only tolerate low volumes of fluids or in whom intravenous access for PN delivery is unavailable. However when delay or failure to establish EN is not complicated by fluid restriction, supplementation with PN should be initiated promptly. The clinical efficacy of such nutritional support modalities along with the routine use of high energy feeds need to be explored in future prospective studies.

In contrast to previous studies which assessed energy and nutrient intake on few selected or random days during PICU stay [12,23] this study recorded nutritional support practices over the patients’ entire length of stay in PICU covering nutritional support data from 887 days. This offers a more comprehensive insight into nutritional support practices and EN provision in patients in PICU and explores patterns associated with the overall intake and advancement of EN support over the course of their admission.

Micronutrients are important for health and in critical care requirements may be higher [24]. Intake of vitamins and mineral was suboptimal and well below the minimal requirements for a large proportion of NSDs. In the short term this may have very little importance particularly in children with a good nutritional status prior to hospital admission. However for those who were already malnourished and more difficult to feed, micronutrient supplementation or development and clinical evaluation of new optimized nutritional feeds with better micronutrient profile for exclusive use in PICU may be needed and their impact on nutritional and clinical outcomes should be explored in future studies.

The exclusion of patients on parenteral nutrition support may be seen as a limitation of the study. However a secondary aim of this study was to explore whether we were able to deliver optimal nutritional support through the enteral route, sparing the use of parenteral nutrition. By doing that we have highlighted patients in whom delivery of optimal EN support is challenging and more targeted feeding protocols and use of PN should be used whenever this is possible. The efficacy of these measures should be explored in the future. We also hypothesised that energy requirements equal those of BMR, instead of measuring gaseous exchange with indirect calorimetry [25] and previous studies have shown large discrepancies between predicted and measured energy requirements. However, even if such facilities were available, the results of the current study suggest that it might have still been challenging to achieve optimal nutritional requirements.

## Conclusions

This study highlights the complexities and challenges of the nutritional management of the critically ill child. It shows that within current multidisciplinary practice, nutritional requirements of healthy children are rarely achieved in paediatric critical care. However, every effort should be made by the nutritional support team to optimise nutritional delivery using every possible resource and when this is possible. Such efforts and better nutritional support practices may be facilitated by increasing nutritional awareness and implementation of local management protocols in routine clinical practice [5-7].

## Competing interests

The authors have no conflicts of interest to declare.

## Authors’ contributions

EG, JM, KD, HA collected the data; EG, KG, DY carried out the data/statistical analysis; EG, KG, JM drafted the manuscript; MD, NS, DY contributed to data interpretation and revised the manuscript. All authors read and approved the final manuscript.

## Pre-publication history

The pre-publication history for this paper can be accessed here:

http://www.biomedcentral.com/1471-2431/14/186/prepub
